# The burden of drug resistance tuberculosis in Ghana; results of the First National Survey

**DOI:** 10.1371/journal.pone.0252819

**Published:** 2021-06-10

**Authors:** Augustina Angelina Sylverken, Alexander Kwarteng, Sampson Twumasi-Ankrah, Michael Owusu, Rejoice Agyeiwaa Arthur, Rexford Mawunyo Dumevi, Louis Adu-Amoah, Nicholas Addofoh, Portia Boakye Okyere, Francisca Dzata, Frank Bonsu, Yaw Adusi-Poku, Katharina Kranzer, Andrew Siroka, Wayne van Gemert, Anna Dean, Ellis Owusu-Dabo

**Affiliations:** 1 Department of Theoretical and Applied Biology, Kwame Nkrumah University of Science and Technology, Kumasi, Ghana; 2 Kumasi Centre for Collaborative Research in Tropical Medicine, Kwame Nkrumah University of Science and Technology, Kumasi, Ghana; 3 Department of Biochemistry and Biotechnology, Kwame Nkrumah University of Science and Technology, Kumasi, Ghana; 4 Department of Statistics and Actuarial Science, Kwame Nkrumah University of Science and Technology, Kumasi, Ghana; 5 Department of Medical Laboratory Technology, Kwame Nkrumah University of Science and Technology, Kumasi, Ghana; 6 National Tuberculosis Control Programme, Ghana Health Service, Accra, Ghana; 7 Supranationale Reference Mycobacterium Laboratory, Borstel, Germany; 8 Global Tuberculosis Programme, World Health Organization, Geneva, Switzerland; 9 Department of Global and International Health, School of Public Health, Kwame Nkrumah University of Science and Technology, Kumasi, Ghana; The University of Georgia, UNITED STATES

## Abstract

Resistance to Tuberculosis drugs has become a major threat to the control of tuberculosis (TB) globally. We conducted the first nation-wide drug resistance survey to investigate the level and pattern of resistance to first-line TB drugs among newly and previously treated sputum smear-positive TB cases. We also evaluated associations between potential risk factors and TB drug resistance. Using the World Health Organization (WHO) guidelines on conducting national TB surveys, we selected study participants from 33 health facilities from across the country, grouped into 29 clusters, and included them into the survey. Between April 2016 and June 2017, a total of 927 patients (859 new and 68 previously treated) were enrolled in the survey. *Mycobacterium tuberculosis* complex (MTBC) isolates were successfully cultured from 598 (65.5%) patient samples and underwent DST, 550 from newly diagnosed and 48 from previously treated patients. The proportion of patients who showed resistance to any of the TB drugs tested was 25.2% (95% CI; 21.8–28.9). The most frequent resistance was to Streptomycin (STR) (12.3%), followed by Isoniazid (INH) (10.4%), with Rifampicin (RIF), showing the least resistance of 2.4%. Resistance to Isoniazid and Rifampicin (multi-drug resistance) was found in 19 (3.2%; 95% CI: 1.9–4.9) isolates. Prevalence of multidrug resistance was 7 (1.3%; 95% CI: 0.5–2.6) among newly diagnosed and 12 (25.0%; 95% CI: 13.6–39.6) among previously treated patients. At both univariate and multivariate analysis, MDR-TB was positively associated with previous history of TB treatment (OR = 5.09, 95% CI: 1.75–14.75, p = 0.003); (OR = 5.41, 95% CI: 1.69–17.30, p = 0.004). The higher levels of MDR-TB and overall resistance to any TB drug among previously treated patients raises concerns about adherence to treatment. This calls for strengthening existing TB programme measures to ensure a system for adequately testing and monitoring TB drug resistance.

## Introduction

Tuberculosis (TB) caused by *Mycobacterium tuberculosis* complex (MTBC) is a global threat. Worldwide, it remains the number one cause of death from a single infectious agent. In 2018, 10 million people were diagnosed with the disease resulting in close to 1.5 million deaths [1]. The number of TB cases with resistance to rifampicin (RR-TB), the most effective first-line drug, was estimated at 558 000 people (range, 483 000–639 000). Of this number, nearly 82% had Multidrug resistance-TB (MDR-TB) caused by MTBC resistant to both rifampicin (RIF) and isoniazid (INH) [2]. MDR-TB poses several challenges similar to those encountered in the pre-chemotherapy era, including the inability to cure TB, excessive mortality and morbidity, uninterrupted transmission resulting in a threat to health care workers, and unsustainably costly treatment [3].

Similar to other countries in sub-Saharan Africa, TB is a major public health problem in Ghana. The recent TB prevalence survey reported a prevalence of smear-positive TB of 111 (95% CI: 76–145) per 100,000 among adult population. The prevalence of bacteriologically confirmed TB was 356 (95% CI: 288–425) per 100,000 population [2]. Several studies have reported the emergence of MDR-TB in Ghana [4–10]. In 2018, the first patients with extensive drug-resistant (XDR) TB defined as MDR-TB with additional resistance to at least one fluoroquinolone and an injectable agent (amikacin, capreomycin, or kanamycin) were identified [6,11]. While these studies emphasize the importance of drug susceptibility testing (DST), they do not provide nationally representative estimates. Such estimates are urgently needed to inform guidelines and policies.

To build evidence or generate data to support decision making, the National TB Programme (NTP) in Ghana performed a National TB Drug Resistance Survey (DRS) following the methodology recommended by the World Health Organization (WHO) to establish nationally representative estimates for drug resistance among newly diagnosed and previously treated TB patients. It was also important to investigate the possible risk factors for TB drug resistance.

## Methods

### Study design and sample size estimation

This first DRS was a nation-wide cross-sectional study using cluster randomised sampling informed by the WHO guidelines and recommendations for conducting national TB surveys [12]. The sample size was based on the number of patients newly diagnosed with sputum-smear positive pulmonary TB in 2013 (n = 11793), an assumed rifampicin resistance (RR) prevalence of 1.7% among this group, a design effect of 2 to account for clustering, inflation of 15% to account for losses of samples for reasons such as insufficient volume or contamination, and a desired absolute precision of 1.2% around the estimate of prevalence. The calculated sample size was, therefore, 1100 newly diagnosed sputum smear-positive pulmonary TB patients.

Since the number of previously treated patients is relatively small, all previously treated patients presenting to the selected diagnostic sites were enrolled until enrollment of new smear-positive cases (29 cases) was completed [12].

### Cluster/Site selection

The number of clusters included in the survey was set at 33 for logistic reasons. These were selected using a probability-proportional-to-size (PPS) approach as per WHO guidelines [12] based on the number of new sputum smear-positive pulmonary TB cases notified in each diagnostic facility in 2013. Sites that diagnosed less than 10 positive smear cases in 2013 were excluded. The target cluster size was 29 new sputum-smear positive patients. The participating sites for the Ghana national TB DRS survey are shown in S1 Table.

### Piloting

A pilot study was conducted between October 2015 and March 2016 to assess the workflow and tools used in the study. A total of 90 smear positive sputum samples collected from seven TB diagnostic sites were included in the pilot. None of the pilot sites were included in the final survey, but they had comparable characteristics to the sites selected for the main study. Workflow piloted included sample transportation and laboratory analysis while all data capturing tools were pretested during this period. A copy of the main questionnaire used for the survey is attached as S1 File.

From April 2016 to June 2017, patients presenting to any of the survey sites/clusters with features of pulmonary TB underwent clinical and sputum examination. At the health facility, two sputa (each with a volume ≥ 3mL); one taken on the spot and the other after one hour, were collected from eligible patients. For patients who were smear-positive, they were included in the study, after which a questionnaire designed for surveys [12] was administered to them. Samples were transported in a cold box using a local transport system and tracked via a designed social media platform to the National TB DRS designated Laboratory, the Kumasi Centre for Collaborative Research in Tropical Medicine (KCCR) at KNUST, for processing [13].

The samples were accompanied by filled questionnaires, specimen transfer forms containing information about the date of sputum collection, participant number, laboratory serial number, and sputum smear-positive quantified results from the examination at the site laboratory.

### Inclusion and exclusion criteria

Adult patients (≥18 years) with signs and symptoms of pulmonary TB, with sputum smear positive by microscopy at the designated cluster were deemed eligible for the study. These included both new and previously treated TB smear-positive patients. On the contrary, new smear-positive patients who had been on TB medications for more than seven days were excluded. Further, children below the age of 18 years were excluded. Cases of extra-pulmonary TB were also excluded.

### Laboratory analysis

**Fig 1:**
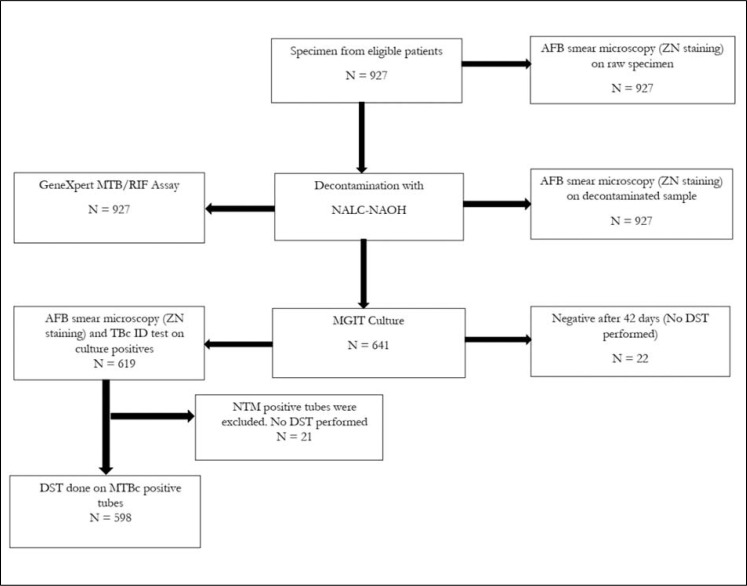
Flow chart describing the bacteriological confirmation of sputum samples received from patients included in the survey (ZN: Ziel Neelson; MTB/RIF: *Mycobacterium tuberculosis*/Rifampicin; NALC-NAOH: N-Acetyl-L-Cysteine-Sodium-Hydroxide; AFB: Acid Fast Bacillus; MGIT: Mycobacterium Growth Indicator Tube; DST: drug susceptibility testing; NTM: Nontuberculous mycobacterial; MTBC: *Mycobacterium tuberculosis* complex.

At the KCCR, samples were re-examined by microscopy using Ziehl-Neelsen staining and graded as either scanty, 1+, 2+, 3+, or negative [14]. The GeneXpert MTB/RIF assay (Cepheid, CA, US) was also performed on the samples [15] at KCCR. All Samples were cultured using the BD BACTEC Mycobacterium Growth Indicator Tube (MGIT) [16]. Briefly, the samples were decontaminated with 4% N-Acetyl-L-Cysteine-Sodium-Hydroxide (NALC-NaOH) and neutralized with 1X phosphate-buffered saline (PBS). Following this, 0.5 ml of the pellet were inoculated in BACTEC™ MGIT 960 tubes (BD Diagnostics, Sparks, MD, USA) at 37°C for 42 days maximum. Phenotypic Drug Susceptibility Test (DST) for rifampicin (RIF), isoniazid (INH), streptomycin (STR), ethambutol (EMB) and pyrazinamide (PZA) were performed with MGIT SIRE and PZA (BD, USA) method on only positive tubes that had MTBC confirmed based on the BACTEC™ MGIT 960 method [16]. *Mycobacterium tuberculosis* strain H37Rv was used as a sensitive control for susceptibility testing. The flow chart for sample processing and laboratory analysis is shown in Fig 1.

### Data management and statistical analysis

Trained health workers used standardized questionnaire in each of the 33 clusters to collect demographic and clinical information from eligible and consented patients including HIV status and previous TB treatment history. These were manually double entered into CSpro (U. S Census Bureau, USA) at KCCR, validated and verified. Smear microscopy, GeneXpert MTB/RIF testing, culture and DST were grouped in Microsoft Excel file and exported into STATA (version 12.0; Stata Corp LP, College Station, TX, USA) for further statistical analysis. *Chi*-square analysis was used to test for significance between risk variables drug resistance. Factors associated with drug-resistant TB were investigated using univariate and multivariate logistic regression models. All statistical tests with Alpha values or *p-*value less than 0.05 (p≤0.05) were deemed significant, and clustering difference was adjusted in the analysis.

### Ethics approval and consent to participate

The study was approved by the Committee for Human Publications, Research and Ethics (CHPRE) of the School of Medical and Dental Sciences, Kwame Nkrumah University of Science and Technology (KNUST) and the Komfo Anokye Teaching Hospital, Kumasi (CHPRE/AP/328/15). We also obtained approval from the Ethics Review Committee of the African Region of the World Health Organization (AFR/ERC/2016/02.01). Written informed consent was also obtained from each participant at the time of recruitment through signatures and thumbprints.

## Results

### Socio-demographic characteristics of the study participants

A total of 927 participants with sputum-smear positive pulmonary TB were enrolled at 33 selected diagnostic sites. Of the 33 sites, eight (8) did not reach target (n = 29). The majority of participants were males 645 (69.6%). The median age for the study subjects was 41 years (interquartile range [IQR]: 18, 22). Less than half of all cases had results of HIV testing available (388/927, 41.9%). Of those with HIV results, 77 were HIV positive (19.8%).

A total 860 (92.8%) of patients enrolled were newly diagnosed cases, while 67 (7.2%) were previously treated cases. Table 1 shows the key socio-demographic characteristics of the patients enrolled in the study. The other socio-demographic is shown in S2 Table.

**Table 1:** Key demographic characteristics of patients enrolled in the National TB drug resistance survey in Ghana in 2017.

Characteristic		Frequency (N = 927)	Percent
**Sex**	Male	645	69.6
	Female	282	30.4
**Age**	18–27	154	16.7
	28–37	216	23.2
	38–47	245	26.4
	48–57	142	15.3
	>57	170	18.4
**HIV result**	Positive	77	8.3
	Negative	311	33.5
	Unknown	539	58.2
**Previous history of TB treatment**	Yes	67	7.2
	No	860	92.8
**Outcome of previous treatment**	Relapse	45	4.9
	Treatment after failure	7	0.7
	Loss to follow-up	12	1.3
	Not applicable	863	93.1

### Microscopy, GeneXpert and culture results

Of the 927 sputum samples investigated, 909 (98.1%), 902 (97.3%), and 598 (64.5%) were positive on repeat smear microscopy in the TB DRS laboratory, Xpert MTB/RIF testing, and cultures, respectively. Of the 902 samples with a positive Xpert MTB/RIF test, 18 (2.0%) were RR; 5 (27.8%) among previously treated patients and 13 (72.2%) among new patients who had never been treated for TB.

### Patterns of TB drug resistance in Ghana

Due to logistical challenges, a total of 595 MTBC isolates underwent DST. The proportion of patients who showed resistance to any TB drugs tested was 25.2% (n = 150/595; 95% CI; 21.8–28.9). The most frequent resistance was to Streptomycin (STR) (12.3%; 73/595), followed by Isoniazid (INH) (10.4%; 62/595) with Rifampicin (RIF) showing the least resistance of 2.4% (14/595).

With respect to any of the TB drugs tested, there was a statistically significant difference between new and previously treated cases (p = 0.041). Here, while 132 (24.1%; 95% CI: 20.6–27.9) of the new cases were resistant to any of the TB drugs, 18 (37.5%; 95% CI: 24.0–52.6) of those who had been previously treated with TB drugs showed resistance (Table 2).

**Table 2:** Patterns of resistance to TB drugs among new and previously treated sputum smear-positive TB patients in Ghana in 2017.

	New cases		Previously Treated			All cases	
Pattern of Resistance	Number (%)	95% CI	Number (%)	95% CI	p-value	Number (%)	95% CI
Total patients	N = 547		N = 48			N = 595	
Susceptible to all	415 (75.9)	72.1–79.4	30 (62.5)	47.4–76.0	0.041	445 (74.8)	71.1–78.2
Any Resistance	132 (24.1)	20.6–27.9	18 (37.5)	24.0–52.6	0.041	150 (25.2)	21.8–28.9
**Any Resistance to;**							
Streptomycin (STR)	64 (11.7)	9.1–14.7	9 (18.8)	8.9–32.6	0.153	73 (12.3)	9.7–15.2
Isoniazid (INH)	53 (9.7)	7.3–12.5	9 (18.8)	8.9–32.6	0.049	62 (10.4)	8.0–13.2
Rifampicin (RIF)	10 (1.8)	0.9–3.3	4 (8.3)	2.3–20.0	0.004	14 (2.4)	1.3–3.9
Ethambutol (EMB)	22 (4.0)	2.5–6.0	3 (6.3)	1.3–17.2	0.461	25 (4.2)	2.7–6.1
Pyrazinamide (PZA)	24 (4.4)	2.8–6.5	3 (6.3)	1.3–17.2	0.552	27 (4.5)	3.0–6.5
**INH + RIF Resistant** (MDR)							
**INH + RIF (only)**	**5 (0.9)**	**0.3–2.1**	**3 (6.3)**	**1.3–17.2**	**0.004**	**8 (1.3)**	**0.6–2.6**
INH + RIF + STR	2 (0.4)	0.0–1.3	3 (6.3)	1.3–17.2	<0.001	5 (0.8)	0.3–2.0
INH + RIF + EMB	0 (0)	-	2 (4.2)	0.5–14.3	<0.001	2 (0.3)	0.0–1.2
INH + RIF + PZA	0 (0)	-	1 (2.1)	0.1–11.1	0.002	1 (0.2)	0–0.9
INH + RIF + STR + EMB	0 (0)	-	2 (4.2)	0.5–14.3	<0.001	2 (0.3)	0–1.2
INH + RIF + STR + PZA	0 (0)	-	1 (2.1)	0.1–11.1	<0.001	1 (0.2)	0–0.9
INH + RIF + EMB + PZA	0 (0)	-	0 (0)	-	-	0 (0)	-
INH + RIF + STR + EMB + PZA	0 (0)	-	0 (0)	-	-	0 (0)	-
**All INH + RIF Resistance**	**7 (1.3)**	**0.5–2.6**	**12 (25.0)**	**13.6–39.6**		**19 (3.2)**	**1.9–4.9**
**INH + Other Resistance**							
INH +STR	11 (2.0)	1.0–3.6	4 (8.3)	2.3–20.0	0.02	15 (2.5)	1.4–4.1
INH + EMB	13 (2.4)	1.3–4.0	2 (4.2)	0.5–14.3	0.153	15 (2.5)	1.4–4.1
INH + PZA	2 (0.4)	0–1.3	1 (2.1)	0–11.1	0.091	3 (0.5)	0.1–1.5
INH + STR + EMB	4 (0.7)	0.2–1.9	2 (4.2)	0.5–14.3	0.078	6 (1.0)	0.4–2.2
INH + STR + PZA	0 (0)	-	1 (2.1)	0–11.1	0.003	1 (0.2)	0–0.9
INH + EMB + PZA	2 (0.4)	0–1.3	0 (0)	-	0.213	2 (0.3)	0–1.2
INH + STR + EMB + PZA	0 (0)	-	0 (0)	-	-	0 (0)	-
**RIF + Other Resistance**							
RIF +STR	2 (0.4)	0–1.3	3 (6.3)	1.3–17.2	<0.001	5 (0.8)	0.3–2.0
RIF + EMB	1 (0.2)	0–1.0	2 (4.2)	0.5–14.3	0.001	3 (0.5)	0.1–1.5
RIF + PZA	0 (0)	-	1 (2.1)	0–11.1	0.002	1 (0.2)	0–0.9
RIF + STR + EMB	0 (0)	-	2 (4.2)	0.5–14.3	<0.001	2 (0.3)	0–1.2
RIF + STR + PZA	0 (0)	-	1 (2.1)	0–11.1	0.003	1 (0.2)	0–0.9
RIF + EMB + PZA	0 (0)	-	0 (0)	-	-	0 (0)	-
RIF + STR + EMB + PZA	0 (0)	-	0 (0)	-	-	0 (0)	-
**Mono Resistance to;**							
Streptomycin (STR)	47 (8.6)	6.4–11.3	5 (10.4)	3.5–22.7	0.668	52 (8.7)	6.6–11.3
Isoniazid (INH)	30 (5.5)	3.7–7.7	5 (10.4)	3.5–22.7	0.164	35 (5.9)	4.1–8.1
Rifampicin (RIF)	4 (0.7)	0.2–1.9	1 (2.1)	0–11.1	0.325	5 (0.8)	0.3–2.0
Ethambutol (EMB)	5 (0.9)	0.3–2.1	1 (2.1)	0–11.1	0.437	6 (1.0)	0.4–2.2
Pyrazinamide (PZA)	14 (2.6)	1.4–4.3	2 (4.2)	0.5–14.3	0.509	16 (2.7)	1.5–4.3
**Other Resistance**							
STR + EMB	5 (0.9)	0.3–2.1	2 (4.2)	0.5–14.3	0.111	7 (1.2)	0.5–2.4
STR + PZA	6 (1.1)	0.4–2.4	1 (2.1)	0–11.1	0.339	7 (1.2)	0.5–2.4
EMB + PZA	5 (0.9)	0.3–2.1	0 (0)	-	0.253	5 (0.8)	0.3–2.0
STR + EMB+ PZA	1 (0.2)	0–1.0	0 (0)	-	0.345	1 (0.2)	0–0.9

As Rifampicin resistance is considered a surrogate marker for MDR-TB, we further estimated the distribution of all RIF resistance among the MTB isolated. Of the 14 (14/595 (2.4%)) RR by Xpert, all were RIF resistant by culture and all of them were MDR-TB. For these, the previously treated cases and new cases accounted for 12 (25.0%; 95% CI: 13.6–39.6) and 7 new (1.3%; 95% CI: 0.5–2.6) respectively. Apart from MDR (INH+RIF) plus STR, we did not observe any of the patients as MDR in addition to any other second or third drug in the new cases. However, in previously treated cases, patients were MDR plus a second drug (either EMB or PZA) or MDR plus EMB and a third drug (STR), or MDR plus PZA and STR. We did not observe MDR in addition to resistance to either EMB and PZA or MDR in addition to EMB, PZA and STR. We also did not observe cases resistant to all the five TB drugs.

We found monoresistance highest for STR with a prevalence of 8.7% (n = 52; 95% CI: 6.6–11.3) and lowest for RIF; 0.8% (n = 5, 95% CI: 0.3–2.0). Though we did not observe a statistical significance, monoresistance to any of the TB drugs was higher in the new TB cases compared to the previously treated cases.

We observed other resistances among the TB patients, mainly the new cases. Patterns included resistance to STR and EMB, STR and PZA, EMB and PZA and resistance to three TB drugs minus RIF and INH (STR, EMB and PZA). All drug resistance patterns detected during the National TB drug resistance survey in Ghana are shown in Table 2.

### Risk factors for drug resistance TB

We further analysed the influence of various factors on drug resistance in this study, the results of which are summarised in Table 3.

**Table 3:** Risk factors for drug resistance TB in Ghana.

Risk Factor		Univariate		Multivariate	
		OR (95% CI)	p-value	OR (95% CI)	p-value
**Sex**	Male	Ref		Ref	
	Female	1.03 (0.36–2.92)	0.95	0.84 (0.25–2.77)	0.77
**Age group (years)**	18–27	Ref		Ref	
	28–47	0.59 (0.13–2.73)	0.50	0.71 (0.14–3.52)	0.67
	>47	0.60 (0.12–3.03)	0.54	0.67 (0.12–3.59)	0.64
**Previous history of TB treatment**	No	Ref		Ref	
	Yes	5.09 (1.75–14.75)	0.003	5.41 (1.69–17.30)	0.004
**Family History of TB**	Yes	Ref		Ref	
	No	1.77 (0.57–5.55)	0.32	1.44 (0.38–5.50)	0.59
	Don’t know	2.3 (0.25–20.99)	0.46	8.66 (0.62–121.77)	0.11
**History of death of family member from TB**	Yes	Ref		Ref	
	No	1.23 (0.16–9.53)	0.85	1.15 (0.11–11.97)	0.91
	Don’t know	0.64 (0.06–6.29)	0.69	0.28 (0.02–4.08)	0.35
**Smoking Status**	Yes currently	ref		Ref	
	No, I’ve never smoked	0.91 (0.11–7.03)	0.92	0.93 (0.10–8.64)	0.95
	No, but I used to smoke	1.49(0.15–14.5)	0.73	1.75 (0.15–19.77)	0.65
	No, but I live with a smoker	1	-	1	-
**Alcohol Use**	Yes	ref		Ref	
	No, I’ve never taken alcohol	1.29 (0.38–4.27)	0.67	1.46 (0.38–5.56)	0.58
	No, but I used to take alcohol	0.79 (0.25–2.55)	0.70	0.76 (0.22–2.63)	0.66

At both univariate and multivariate analysis, MDR-TB was positively associated with previous history of TB treatment (OR = 5.09 (1.75–14.75, p = 0.003), (OR = 5.41, 95% CI: 1.69–17.30, p = 0.004). In contrast, HIV status and sex of patients and the other variables had no association with drug resistance TB (p>0.05).

## Discussion

This study is the first national representative TB drug resistance survey in Ghana and one of the studies done in sub-Saharan Africa to estimate the burden of resistance to selected TB drugs on a national scale. We estimated an overall prevalence of 25.2% resistance to any of the five TB drugs in Ghanaian patients. We also detected an overall multi-drug resistance (MDR) prevalence of 3.2%, with a rate of 1.3% and 25.0% among new and previously treated patients, respectively. Globally, 3.5% of new TB cases and 18% of previously treated cases have been notified to have MDR TB [17]. With the detected MDR rate during the survey, we are tempted to conclude that MDR-TB prevalence among new TB patients in Ghana is low. This is because settings with an MDR-TB prevalence of less than 3% among new patients are classified as having a low MDR-TB burden [18]. On the contrary, MDR-TB among previously treated TB cases was relatively high (25.0%). Other nation-wide surveys in the sub-region have observed varying levels. For example, in Uganda, lower rates of 1.4% and 12.1% among new and previously treated patients were recorded during their national TB drug-resistant survey [17]. In nearby Burkina Faso, levels of 3.4% in new cases similar to what we detected, but very high levels in previously treated patients (50.5%) have been reported [19]. Similarly, in Côte d’Ivoire, the proportion of patients with rifampicin resistance was estimated to be 4.6% (95% CI: 2.4–6.7) and 22% (95% CI: 13.7–30.3), respectively, for new and previously treated patients [20]. In Tanzania, the prevalence of any resistance among new and previously treated patients was 8.3% and 20%, respectively [21]. A recent review has reported a pooled prevalence of 2.1% MDR-TB in new patients in sub-Saharan Africa [22] with the same level as observed in this survey in Kenya (1.3%) [23], and levels as high as 5.2% in Somalia [24], and much higher levels of 17.6% in Nigeria [25].

There seems to be limited information on the prevalence of MDR-TB in new and previously treated TB patients in Ghana. The few studies available have been limited to previously treated patients and have recorded reported pan resistance levels between 17.9% [6] and 83% [26] among chronic TB patients from a teaching hospital. A large study conducted in two large regions in Ghana between 2000–2004 recorded an overall primary drug resistance prevalence of 23.5% [4]. This is very similar to the overall primary drug resistance of 25.2% detected during the national drug resistance survey amongst TB patients in Ghana. Even though both studies used different diagnostic methods, and of course, the previous study was limited in terms of nation-wide coverage, the similarity in terms of the burden of resistance to any of the TB drugs is quite surprising. On the contrary, a more recent study aimed at establishing the prevalence of human immunodeficiency virus (HIV) and TB in Ghana did not observe any MDR among the TB patients; a finding the authors attributed to the inclusion of mainly new TB patients in their study [27]. Only with the conduct of a national survey can we find the true burden of MDR-TB to enable policy makers to chart suitable paths towards the management of drug-resistant TB. Thus, the MDR level of this national survey is representative of the entire country. To this end, the data from this first Ghana TB DR national survey show that while MDR among newly diagnosed smear-positive TB patients is low, the level among previously treated cases is high (which is usually the case). Apart from an efficient NTP, this low detection of MDR among treatment naïve patients can potentially be attributed to the Directly Observed Treatment Short-course system in Ghana since the 1980s. This system adopts judicious use of rifampicin only during the first 2 months (2EHRZ/6EH) for new TB cases that are known to contribute over 90% of the disease burden. Despite reports indicating the failure/non-adherence of the DOTS strategy [28], low rates of initial drug resistance have been reported in countries where the DOTS has been successfully implemented. This gives a hint that adequate use of standardized treatment regimens under DOTS may potentially limit further emergence of drug resistance. However, whether this will substantially reduce the current degree of resistance observed in Ghana, especially in new TB cases, needs to be evaluated. The relatively high degree of mono-resistance to Streptomycin and Isoniazid and the seemingly low level to Rifampicin (RIF) observed in the survey has been reported in Ghana [4–6,9]. Since RIF is used as a surrogate for MDR-TB on GeneXpert, and importantly, this is what drives patient management and treatment regiments, this trend, in terms of resistance to TB drugs especially to Streptomycin and Isoniazid in Ghana calls for attention. There is no gainsaying that with the increasing use of GeneXpert for simultaneous detection of TB and resistance to RIF, a growing number of RIF resistant-TB cases (without further testing for isoniazid resistance) are being detected and notified. However, one is unsure whether GeneXpert detection of low MDR-TB (RIF resistant—RR) would imply low resistance to other TB drugs. This has important implications if GeneXpert is used as a proxy for detection of MDR-TB. Under such circumstances, most INH-mono-resistant cases would obviously not be detected and may be treated as susceptible to first-line regimen containing INH. This can render the first-line regimen ineffective, especially in previously treated patients. It is, however, gratifying that the WHO has a special treatment regimen recommended for such patients.

Usually, once RR-TB is detected by GeneXpert, health workers are expected to conduct cultures and DSTs to ensure resistance patterns to the other anti-TB drugs. To this end, for all the samples, we observed very high concordance between detection by smear microscopy, MTB detection, and RR by GeneXpert and phenotypic DST. However, we are careful in exaggerating the superiority of one diagnostic method over the other bearing in mind that all patients included in the survey had to be positive before being included in the survey. This notwithstanding, the National TB control program in Ghana has recently scaled up the use of GeneXpert for the detection of MDR (RR) TB. A major advantage of this move is that this will allow for the rapid initiation of treatment while awaiting culture and DST. While this strategy is recommended by the WHO [29], second-line drugs and culture and DSTs are not always readily available in Ghana. There are only five TB laboratories that have been equipped to perform culture and phenotypic DSTs. With such a limited number of laboratories and other challenges, culture and DSTs may not be performed on GeneXpert MTB/RIF detected RR. Additionally, transportation challenges, poor quality of specimens, specimens that are never collected or because the patient was not tracked may render culture and phenotypic DSTs ineffective thereby potentially contributing to the spread of the disease. The allocation of resources to detect and treat MDR-TB in low-resource settings remains controversial [30].

WHO recommends universal DST for at least RR as part of the End TB Strategy [31], but whether this is adhered to is another issue. Whereas some advocate that priority is given to the effective treatment of drug sensitive disease, thus preventing the emergence of drug-resistance [32], others argue that drug resistant cases should be detected and treated both for the good of the individual and to reduce ongoing transmission of drug resistant disease [33]. Indeed, control of drug resistant tuberculosis requires a strong health infrastructure to ensure testing of samples, the delivery of effective therapy coupled with surveillance and monitoring activities. These would, in turn, enable timely intervention to limit transmission and spread of the disease.

The higher rates among previously treated TB patients, as we saw in this study, have been attributed to the stepwise selection of mutants due to drug resistance-conferring genes [34]. Apart from this gene conferring theory, such high levels of MDR-TB (25.0%) and resistance to any drug (37.5%) among previously treated patients raises concerns about adherence to treatment. While poor quality anti-TB drug prescriptions have virtually been eliminated in Ghana, some incidents of poor case management related to adherence, may partly explain the high level of emergence of drug resistance TB among previously treated TB patients. In the case of Rifampicin, apart from the TB control program that uses it for the management of TB, its use is very restricted in Ghana. On the contrary, there seem to be a high detection of Streptomycin resistance during the survey as observed in previous studies [4,6]. With such high rates, it is not surprising that the Ghana national Tuberculosis Control Programme has since several years ago removed Streptomycin from the list of antiTB medication because of ototoxicity.

Our results on possible risk factors of MDR TB indicated previous treatment as the strongest determinant. The high risk associated with previous treatment implies that the common practice of re-treating TB cases with first-line drugs may generally be ineffective in Ghana. Thankfully, the NTP in Ghana has halted this practise a couple of years ago. Several studies have also shown previous drug treatment as the strongest determinant of MDR-TB [35–37]. Depending on the country, it is known that the prevalence of MDR-TB in retreatment cases is between 30% and 80% [38]. Since this is yet to be reported for Ghana, a concerted effort is needed in coming up with a possible revised treatment regimen for patients with history of TB treatment. Added to this will be an uninterrupted supply of second-line drugs and a robust system that ensures rapid testing for drug resistance for all patients with TB. This may warrant further studies in other aspects of treatment such as the drugs used and the length of treatment as these may contribute to improving control programmes.

## Limitations

Our survey has some limitations. Firstly, phenotypic DST was not performed for nearly half of the patients, and so we are careful in extrapolating the results to the entire study population. Secondly, while there are several private hospitals in Ghana, this survey only represented patients diagnosed through the NTP supervised TB diagnostic facilities. These private laboratories have policies for referral which indicates the need to refer TB cases to the public sector. The weakness is that this system is not well supervised. As such, it does not account for drug resistance patterns among TB patients who do not have access to these health systems. Thirdly, although the survey was conducted using the most recent WHO guidance [12], smear-negative patients were not included in the survey. However, there is no evidence for different drug resistance patterns among smear-negative TB patients. Further, the sampling frame for this survey was based on TB case notification in 2013 in Ghana. A number of changes in the healthcare delivery system, such as the deployment of several GeneXpert machines, the establishment of new regions and districts, and new health facilities, which did not make part of the sampling frame but shared the patients with the included facilities. Despite these limitations, our results highlight the urgent need for efforts to address drug-resistant TB, especially the use of anti-TB drugs (Streptomycin) in Ghana.

## Conclusions and lessons learnt

This first Ghana nation-wide TB drug resistance survey has provided compelling evidence that the prevalence of RR-TB in Ghana is relatively low. However, we estimated a relatively high burden of MDR-TB among previously treated patients. This will require, among other things, improvements in both overall detection and coverage of diagnostic DST. This means that Ghana needs to establish a continuous surveillance system based on universal DST for at least RIF. Further, strengthening laboratory capacity and wider introduction and uptake of new rapid diagnostics such as Line Probe Assays and testing for second-line drugs need to be incorporated into existing TB diagnostic systems. Moreover, patient adherence to first-line drug treatment may need strengthening. Active and frequent monitoring of TB drug resistance is necessary throughout the country, including the non-NTP regulated sectors, using routine surveillance.

This survey has improved the national laboratories’ proficiency in undertaking culture and DST (first-line). This is expected to increase patient coverage of DSTs in Ghana, including previously treated TB patients who, as in many other countries, harbour a substantial part of the MDR-TB caseload. Further, the current ongoing expansion in the use of GeneXpert MTB/RIF nation-wide in Ghana is expected to improve access to patient testing.

We, therefore, conducted a population-based TB drug resistance survey in 2016 with the National Tuberculosis Control Programme (NTP) as Supervisors. The Kumasi Centre for Collaborative Research in Tropical Medicine (KCCR), based at the KNUST as implementors, the Chest Clinic of the Korle Bu Teaching Hospital in Accra as the National Reference Laboratory and the National Reference Laboratory for Mycobacteria in Borstel, Germany as the Supra Reference laboratory (SRL).

## Supporting information

S1 Table Names of facilities for TB DRS. A list of participating hospitals selected for the Ghana National Tuberculosis Control programmme showing districts and regions.
(PDF)

S2 Table Demographic information. Demographic characteristics of patients enrolled in the National TB drug resistance survey in Ghana in 2017.
(PDF)

S1 File Screening questionnaire for TB DRS. TB Drug Resistance Survey in Ghana Screening questionnaire.
(PDF)

## Abbreviations

TB: Tuberculosis
DR: Drug-Resistant
DRS: Drug resistance survey
XDR: Extensively drug-resistant tuberculosis
WHO: World Health organization
KCCR: Kumasi Centre for Collaborative Research in Tropical Medicine
DOTS: Directly Observed Treatment Short-course (DOTS)
NTP: National Tuberculosis Control Programme
SRL: Supra Reference laboratory
AFB: Acid Fast Bacilli
MGIT: Mycobacterium Growth Indicator Tube
NALC-NaOH: N-Acetyl-L-Cysteine-Sodium-Hydroxide
DST: drug susceptibility test
RIF: Rifampicin
INH: Isoniazid
STR: Streptomycin
EMB: Ethambutol
PZA: Pyrazinamide
MTBC: *Mycobacterium tuberculosis* complex
CHPRE: Committee for Human Publications, Research and Ethics
GPRTU: Ghana Private Road Transport Union
