# Supplementary material for: The burden of drug resistance tuberculosis in Ghana; results of the First National Survey
Source: PLoS One. 2021 Jun 10;16(6):e0252819. doi: 10.1371/journal.pone.0252819 (PMC8191906; doi:10.1371/journal.pone.0252819)
Supplement: S1 Table — A list of participating hospitals selected for the Ghana National Tuberculosis Control programmme showing districts and regions. (PDF) [file pone.0252819.s001.pdf]

S1 Table: A list of participating hospitals selected for the Ghana National Tuberculosis Control programmme showing districts and regions

Diagnostic Centers	Districts	Regions
Effiduase Government Hospital	Juaben	Ashanti
Manhyia Government Hospital	Kumasi metro	Ashanti
Komfo Anokye Teaching Hospital	Kumasi metro	Ashanti
Bawjiase Health Centre	Awutu Senya	Central
Worawora Government Hospital	Biakoye	Volta
Hohoe Municipal Hospital	Hohoe	Volta
Battor Government Hospital	North Tongu	Volta
Asamankese Government Hospital	West Akim	Eastern
Nsawam Government Hopital	Nsawam Adoagyri	Eastern
Tetteh Quarshie Memorial Hospital	Akwapim North	Eastern
Holy Family Hospital	Kwahu West	Eastern
Regional Hospital (Eastern Region)	New Juaben	Eastern
Kintampo Municipal Hospital	Kintampo North	Brong Ahafo
Techiman Holy Family Hospital	Techiman South	Brong Ahafo
Lawra District Hospital	Lawra	Upper West
Nalerigu District Hospital	East Mamprusi	Northern
Salaga District Hospital	East Gonja	Northern
Bongo District Hospital	Bongo	Upper East
Bawku Presbyterian Hospital	Bawku Municipal	Upper East
St. Martins de Porres Hospital Eikwe	Ellembele	Western
Wassa Akropong Government Hospital	Wassa Amenfi East	Western
Tarkwa Government Hospital	Tarkwa Municipal	Western
Tema General Hospital	Tema Metropolitan	Greater Accra
Kaneshie Polyclinic	Accra Metro	Greater Accra
Madina Polyclinic, Kekele	Ga East Municipal	Greater Accra
Achimota Government Hospital	Accra Metro	Greater Accra
Tema Polyclinic	Tema Metropolitan	Greater Accra
Saltpond Municipal Hospital	Mfantsiman	Central
Abura Dunkwa Government Hospital	Abura Asebu Kwamankese	Central
Swredu Government Hospital	Agona West	Central
Cape Coast Teaching Hospital	Cape Coast	Central
Dunkwa on offin Government Hospital	Upper Denkyira East	Central
Effia Nkwanta Regional Hospital	Sekondi Takoradi	Western
