# Supplementary material for: The burden of drug resistance tuberculosis in Ghana; results of the First National Survey
Source: PLoS One. 2021 Jun 10;16(6):e0252819. doi: 10.1371/journal.pone.0252819 (PMC8191906; doi:10.1371/journal.pone.0252819)
Supplement: S2 Table — Demographic characteristics of patients enrolled in the National TB drug resistance survey in Ghana in 2017. (PDF) [file pone.0252819.s002.pdf]

S2 Table: Demographic characteristics of patients enrolled in the National TB drug resistance survey in Ghana in 2017

**Characteristic**		**Frequency (N = 927)**	**Percent**
**Sex**	Male	645	69.6
	Female	282	30.4
**Age**	18-27	154	16.7
	28-37	216	23.2
	38-47	245	26.4
	48-57	142	15.3
	>57	170	18.4
**HIV result**	Positive	77	8.3
	Negative	311	33.5
	Unknown	539	58.2
**Previous history of TB treatment**	Yes	67	7.2
	No	860	92.8
**Outcome of previous treatment**	Relapse	45	4.9
	Treatment after failure	7	0.7
	Loss to follow-up	12	1.3
	Not applicable	863	93.1
**Region of Hometown**	Ashanti region	73	7.9
	Greater Accra region	44	4.8
	Central region	218	23.5
	Western region	121	13.1
	Eastern region	118	12.7
	Volta region	136	14.7
	Brong Ahafo region	33	3.6
	Northern region	83	8.9
	Upper East region	47	5.1
	Upper West region	48	5.2
	Other regions outside Ghana	6	0.6
**Occupation**	Public servant	48	5.2
	Trader	169	18.3
	Farmer	240	25.9
	Mining	37	4

	Unemployed	104	11.2
	Artisan	164	17.7
	Student	43	4.6
	Pensioner	18	1.9
	Fisherman	29	3.1
	Other	75	8.1
**Highest level of education**	None	218	23.5
	Non formal	22	2.4
	Primary	145	15.6
	JHS/Middle School	357	38.5
	SHS/Technical/Vocational	147	15.9
	Tertiary	38	4.1
**Religion**	Christian	705	76.1
	Islam	114	12.3
	Traditional	54	5.8
	None	51	5.5
	Other	3	0.3
**Marital status**	Single/ Never Married	259	28
	Married	412	44.5
	Separated	42	4.5
	Divorced	108	11.6
	Widow/widower	80	8.6
	Co-habitation	26	2.8
**Type of housing**	Separate house	150	16.2
	Semi-detached house	83	8.9
	Flat/apartment	66	7.1
	Compound house	559	60.3
	Huts/buildings, same compound	32	3.5
	Huts/buildings, separate compounds	11	1.2
	Improvised home (kiosk, container)	13	1.4
	Living quarter attached to office/shop	3	0.3
	Uncompleted building	6	0.7
	Other	4	0.4
**Lives alone**	Yes	174	18.8
	No	753	81.2
**Prison History**	Yes	94	10.1

	No	833	89.9
**Family History of TB**	Yes	131	14.1
	No	717	77.4
	Don't know	79	8.5
**History of death of family member from TB**	Yes	52	5.6
	No	780	84.1
	Don't know	95	10.3
**Smoking Status**	Yes currently	53	5.7
	No, I've never smoked	630	68
	No, but I used to smoke	230	24.8
	No, but I live with a smoker	14	1.5
**Alcohol Use**	Yes	249	26.9
	No, I've never taken alcohol	397	42.8
	No, but I used to take alcohol	281	30.3
