# Supplementary material for: The burden of drug resistance tuberculosis in Ghana; results of the First National Survey
Source: PLoS One. 2021 Jun 10;16(6):e0252819. doi: 10.1371/journal.pone.0252819 (PMC8191906; doi:10.1371/journal.pone.0252819)
Supplement: S1 File — TB Drug Resistance Survey in Ghana Screening questionnaire. (PDF) [file pone.0252819.s003.pdf]

S1 File:

TB Drug Resistance Survey in Ghana

SCREENING QUESTIONNAIRE

PLEASE WRITE IN CAPITAL LETTERS

INTERVIEW INFORMATION

NB: Please all responses must be filled. Also please write "X" in all skipped responses.

A

A1. Patient ID:

A2. DATE OF INTERVIEW (dd/mm/yyyy)		
A3. INTERVIEWER'S NAME		
A4	**NB: Fill in reason when ans 2 – 5 is selected**	
RESULT CODES:	1. Completed<br>2. Partly Completed<br>3. Postponed<br>4. Refused<br>5. Others	
A5. Other (Specify)		
A6. Reason		

A7. Surname	A8. First Name	Date of Birth
		A9 |__|__| DD<br>A10 |__|__| MM<br>A11 |__|__||__|__| YYYY
A12. Age in completed years	A13. Close contact's Name	A14. Close Contact's active Phone No
A15. Participant's active Phone Number:	A16. House No	A17. Town (Residence)
A18. District (Residence)	A19. Region (Residence)	A20. Traceable Address/or landmark
A21. If already a registered TB patient, registration number	A22. If already a registered TB patient, treatment category	A23. If already a registered TB patient, last smear result

|__|__|__|__|__|__|__|__|__|	|__|__|__|__|__|__|__|__|__|	|__|__|__|__|__|__|__|__|__|

INTERVIEWER: INTRODUCTION AND CONSENT. May I begin the interview now?

NO	QUESTIONS AND FILTERS	CODING CATEGORIES	RESPONSE CODES ONLY
**B - Personal Information**<br>I would like to start by asking you a few questions about yourself			
B1	Consent	1. Yes<br>2. No	
B2	Where do you come from (Hometown)?		
B3	In which Region is your Hometown?		
B4	Sex	1. Male<br>2. Female	
B5	To which ethnic group do you belong?		
B6	Occupation?	1. Public servant<br>2. Trader<br>3. Farmer<br>4. Mining<br>5. Unemployed<br>6. Artisan<br>7. Student<br>8. Pensioner<br>9. Fisherman<br>10. Other	
B7	Other (Specify)		
**C - Screening for symptoms**			
C1	Do you cough currently?<br>**If No ➔C3**	1. Yes<br>2. No	
C2	If Yes, for how long in weeks?	1. Less than two weeks<br>2. 2 – 4 weeks<br>3. More than 4 weeks	
C3	Do you cough up sputum?<br>**If No ➔C7**	1. Yes<br>2. No	
C4	If Yes, for how long in weeks?	1. Less than two weeks<br>2. 2 – 4 weeks<br>3. More than 4 weeks	
C5	If Yes, do you cough blood stained sputum?<br>**If No ➔C7**	1. Yes<br>2. No	
C6	If Yes, for how long in weeks?	1. Less than two weeks<br>2. 2 – 4 weeks	

		3. More than 4 weeks	
C7	Do you currently have chest pain?<br>**If No ➔C9**	1. Yes<br>2. No	
C8	If Yes, for how long in weeks?	1. Less than two weeks<br>2. 2 – 4 weeks<br>3. More than 4 weeks	
C9	Have you experienced any unintentional or unexpected weight loss in the past month?<br>**If No ➔C11**	1. Yes<br>2. No	
C10	If Yes, for how long in weeks?	1. Less than two weeks<br>2. 2 – 4 weeks<br>3. More than 4 weeks	
C11	Do you currently have Fever?<br>**If No ➔C13**	1. Yes<br>2. No	
C12	If Yes, for how long in weeks?	1. Less than two weeks<br>2. 2 – 4 weeks<br>3. More than 4 weeks	
C13	Do you have night sweats?<br>**If No ➔C15**	1. Yes<br>2. No	
C14	If Yes, for how long in weeks?	1. Less than two weeks<br>2. 2 – 4 weeks<br>3. More than 4 weeks	
C15	Did you have the same/similar symptoms prior to this episode of illness?	1. Yes<br>2. No	
**D - TB status and treatment history**			
D1	Have you had a sputum examination before?	1. Yes<br>2. No<br>3. Don't remember	
D2	Have you ever been previously diagnosed with TB?<br>(Please go through available medical files and other documents) **If No/Don't remember ➔ D4**	1. Yes<br>2. No<br>3. Don't remember	
D3	If Yes, how long ago have you been diagnosed as having TB?	1. 1 - 6 months<br>2. 7 - 12 months<br>3. 1 - 3 years<br>4. More than 3 years	
D4	Have you ever taken anti-TB drugs for more than a month?<br>**If No ➔D7**	1. Yes<br>2. No<br>3. Don't know	
D5	How many episodes of treatment have you had?	1. Once<br>2. Twice<br>3. Other	
D6	Other (Specify)		
D7	Have you ever previously received injections for more than one month?	1. Yes<br>2. No<br>3. Don't Know	

D8	Are you currently taking any anti-TB medication?<br>**If No ➔D24**	1. Yes<br>2. No<br>3. Don't know	
D8a	If Yes, where did/do you get your medication from?	1. Hospital pharmacy<br>2. Community pharmacy<br>3. Licensed Chemical seller<br>4. Other	
D9	Other (specify)		
D10	Has there been a day that you forgot to take your medication?<br>**If No ➔D12**	1. Yes<br>2. No	
D11	If Yes, for approximately how long?	1. One day<br>2. 2-3 days<br>3. More than a week<br>4. More than a month	
D12	Have you ever run out of your medication?	1. Yes<br>2. No	
D13	Do you sometimes take your medication late?	1. Yes<br>2. No	
D14	Has there ever been a reason not to take your medication?<br>**If No ➔D16**	1. Yes<br>2. No	
D15	If Yes, what are some of the reasons?		
D16	Do you think that you have too many pills/tablets to take/swallow?	1. Yes<br>2. No	
D17	About how many times in the past month have you missed a whole day of treatment?	1. Once<br>2. Twice<br>3. Thrice<br>4. More than three times<br>5. Never<br>6. Don't remember	
D18	Is it easy to obtain your TB medications?<br>**If No ➔D20**	1. Yes<br>2. No	
D19	If Yes, do/did you have problem in obtaining the anti-TB medicines?	1. Yes<br>2. No	
	**Please tell us some of the problems you face**		
D20	Money for transport	1. Yes<br>2. No	
D21	Money for drugs	1. Yes<br>2. No	
D22	Lack of TB medicine from stores	1. Yes<br>2. No	
D23	Others		
D24	Do you take other medications for your TB treatment?<br>**If No ➔E1**<br>**If Yes ➔D25**	1. Yes<br>2. No	
	**If Yes, what are your other sources of medication apart from hospital and pharmacy?**		

D25	Herbal medicines	1. Yes<br>2. No	
D26	Chinese medicines	1. Yes<br>2. No	
D27	Homeopathy	1. Yes<br>2. No	
D28	Acupuncture	1. Yes<br>2. No	
D29	Traditional medicines	1. Yes<br>2. No	
D30	Prayer camp	1. Yes<br>2. No	
D31	Other (specify)		
**E - Final decision**			
E1	Has the patient been treated for more than one month<br>**If No /Unknown➔F1**	1. Yes<br>2. No<br>3. Unknown	
E2	If Yes, what was the outcome of the previous treatment?	1. Cured/treatment completed<br>2. Failed new patient regimen of first-<br>line drugs only<br>3. Failed re-treatment regimen of<br>first-line drugs only<br>4. Failed regimen which included second-line drugs<br>5. Lost to follow-up<br>6. Other<br>7. Unknown	
E3	Other (Specify)		
**F - Demographics**			
F1	What is your highest level of education	1. None<br>2. Non formal<br>3. Primary<br>4. JHS/Middle School<br>5. SHS/Technical/Vocational<br>6. Tertiary	
F2	Religion	1. Christian<br>2. Islam<br>3. Traditional<br>4. None<br>5. Other	
F3	Other (specify)		
F4	Marital status	1. Single/ Never Married<br>2. Married<br>3. Separated<br>4. Divorced	

		5. Widow/widower<br>6. Co-habitation	
F5	Have you had an HIV test before?<br>**If No ➔G1**	1. Yes<br>2. No	
F6	What is your HIV status? (Please go through available documents and crosscheck)	1. Positive<br>2. Negative<br>3. Indeterminate<br>4. Unknown	
**G - Housing**			
G1	What type of housing do you live in?	1. Separate house<br>2. Semi-detached house<br>3. Flat/apartment<br>4. Compound house<br>5. Huts/buildings, same compound<br>6. Huts/buildings, separate compounds<br>7. Tents<br>8. Improvised home (kiosk, container)<br>9. Living quarter attached to office/shop<br>10. Uncompleted building<br>11. Other	
G2	Other (specify)		
G3	Do you live alone?<br>**If Yes ➔G5**	1. Yes<br>2. No	
G4	If No, how many people do you share accommodation with?	1. With one other person<br>2. 2 - 5 other persons<br>3. 6 - 10 other persons<br>4. More than 10 persons	
G5	Have you ever been imprisoned?<br>**If No ➔H1**	1. Yes<br>2. No	
G6	If Yes, Have you been imprisoned in the last 5 years?	1. Yes<br>2. No	
**H - Family history with TB infection**			
H1	Has anyone in your immediate family (your parents, brothers, sisters, or children) ever been diagnosed with TB? **If No/ Don't know ➔H4**	1. Yes<br>2. No<br>3. Don't know	
H2	If Yes, which family member/s?	1. Father<br>2. Mother<br>3. Brother<br>4. Sister<br>5. Son<br>6. Daughter<br>7. Other	

H3	Other (specify)		
H4	Has anyone in your immediate family (your parents, brothers, sisters, or children) been declared dead from TB disease? **If No/Don't know ➔I1**	1. Yes<br>2. No<br>3. Don't know	
H5	If Yes, which family member/s?	1. Father<br>2. Mother<br>3. Brother<br>4. Sister<br>5. Son<br>6. Daughter<br>7. Other	
H6	Other (specify)		
	**I - Lifestyle**		
I1	Do you smoke?	1. Yes currently **➔I2➔ I4**<br>2. No, I've never smoked **➔ I5**<br>3. No, but I used to smoke **➔I3➔ I5**<br>4. No, but I live with a smoker **➔ I5**	
I2	How long have/did you smoke(d)?	Year……………..<br>Month………….<br>Weeks…………….	
I3	How long has it been since you quit smoking?	Year…………………<br>Month………………<br>Weeks………………	
I4	If Yes, currently, how many sticks do you smoke a day?	- - /day	
I5	Do you take alcohol?<br>**If I1 = No, I've never taken alcohol ➔J1**	1. Yes<br>2. No, I've never taken alcohol<br>3. No, but I used to take alcohol	
I6	If **Yes, or No, but I used to take alcohol**, on average how often do/did you drink alcohol?	1. At least once per day (one drink)<br>2. At least once per week<br>3. At least once per month<br>4. At least once per year	
	**J - Comorbidity screening**		
	**Have you ever been diagnosed with any of the following conditions?**		
J1	Hypertension	1. Yes<br>2. No	
J2	Diabetes	1. Yes<br>2. No	
J3	Asthma	1. Yes<br>2. No	

J4	Cancer	1. Yes<br>2. No	
J5	Cancer (Specify)		
J6	When was the first time you were told that you had this condition?	1. Less than one year<br>2. Between 1-3 years<br>3. 4-6 years<br>4. More than 6 years<br>5. None	

K - FOR OFFICIAL USE ONLY

Medical records

K1	After going through available medical documents, have you discovered that the patient has been registered for TB treatment before?	1. Yes<br>2. No<br>3. Unknown<br>4. No available records	
	**INTERVIEWER:** THANK THE RESPONDENT FOR COMPLETING THIS FIRST PART OF THE INTERVIEW.		

	**INTERVIEWER:** Check the records to confirm current status of patient.		
K2	New smear positive<br>**If Yes ➔K5**	1. Yes<br>2. No	
K3	Previously treated	1. Relapse<br>2. Treatment after failure<br>3. Loss to follow-up<br>4. Others	
K4	Others (undocumented information)		
	**Specimen taken:**		
K5	Sputum for TB DR test	1. Yes<br>2. No	
K6	Blood for genetics test	1. Yes<br>2. No	
K7	Blood for immunology test	1. Yes<br>2. No	

L - INTERVIEWER'S REMARKS

L1	**Was anyone else present during the interview?**	1. No one else was present<br>2. Respondent's doctor/nurse<br>3. Other clients/Patients<br>4. Respondent's relative<br>5. Other Health professionals	
L2	**In general, what was the respondent's attitude during the interview?**	1. Friendly, interested<br>2. Was cooperative, but not particularly interested<br>3. Impatient, worried<br>4. Hostile	

L3	**Did the respondent understand the questions?**	1. Understood well<br>2. Did not understand very well<br>3. Understood poorly	
